# Temporal gene expression patterns in the coral *Euphyllia paradivisa* reveal the complexity of biological clocks in the cnidarian-algal symbiosis

**DOI:** 10.1126/sciadv.abo6467

**Published:** 2022-09-16

**Authors:** Mieka Rinsky, Eviatar Weizman, Hiba Waldman Ben-Asher, Gal Eyal, Bokai Zhu, Oren Levy

**Affiliations:** ^1^ Mina and Everard Goodman Faculty of Life Sciences, Bar-Ilan University, Ramat Gan 52900, Israel.; ^2^ ARC Centre of Excellence for Coral Reef Studies, School of Biological Sciences, University of Queensland St. Lucia, Queensland 4072, Australia.; ^3^ Aging Institute of UPMC, University of Pittsburgh School of Medicine, Pittsburgh, PA, USA.; ^4^ Division of Endocrinology and Metabolism, Department of Medicine, University of Pittsburgh School of Medicine, Pittsburgh, PA, USA.

## Abstract

Studying chronobiology in reef-building corals is challenging due to the tightly coupled symbiosis with their photosynthetic algae, Symbiodiniaceae. Although symbiosis requires metabolic synchronization and coordination of cellular processes in the holobiont, the cross-talk between the host and symbiont’s clocks is still puzzling. Here, we use the mesophotic coral *Euphyllia paradivisa* to examine temporal gene expression patterns in symbiotic and aposymbiotic morphs exposed to natural light/dark cycles and constant darkness. Our comparative transcriptomic analyses revealed circadian and circatidal cycles of gene expression with a predominant diel pattern in both coral morphs. We found a substantial number of transcripts consistently rhythmic under both light conditions, including genes likely involved in the cnidarians’ circadian clock, thus indicating that an endogenous clock, which can oscillate independently from the Symbiodiniaceae clock, exists in *E. paradivisa*. The analysis further manifests the remarkable impacts of symbiosis on transcriptional rhythms and implies that the algae’s presence influences the host’s biorhythm.

## Teaser

The symbiotic state of the coral host affects diurnal gene expression and circadian regulation.

## INTRODUCTION

The solar, tidal, and lunar cycles significantly affect organisms inhabiting the marine ecosystem in general and coral reefs in particular (*1*). Anticipating such environmental cycles has favored the development of endogenous biological clocks, which enhance the organism’s fitness and survival by timing different biological processes (*2*, *3*). Studying biological rhythms has focused mainly on circadian clocks, a molecular mechanism based on transcription-translation feedback loops (TTFLs) that govern circadian (~24-hour) rhythmicity (*4*). While this mechanism is shared among all kingdoms of life, the specific clock components involved in the establishment of the TTFL and their interactions differ between taxa (*5*). Behavioral and molecular studies conducted on different marine organisms have shown an additional biological rhythm of ∼12 hours that is governed by an endogenous circatidal clock (*6*–*9*). The 12-hour rhythms of gene expression are conserved across divergent species indicating potential evolutionary conservation of the circatidal clock (*10*). Nevertheless, in contrast to the wealth of information about the circadian clock, the molecular components of circatidal clocks and the environmental cues that synchronize tidal rhythms are poorly understood. Therefore, questions regarding the interaction between the two clocks, particularly whether they share common components or operate independently, remain unresolved (*9*, *11*–*13*).

Within marine ecosystems, coral reefs are an essential reservoir for biological diversity and complexity, appointing their ecological importance (*14*). Their evolutionary success relies on the mutualistic coral-algae symbiosis (*15*). This association further complicates rhythmic behaviors since the circadian photosynthetic cycles, imposed by the symbiotic algae, affect oxygenation and nutrient state in the host tissue (*16*). Although there are some indications for rhythmic and synchronized behaviors between the symbionts (*17*–*19*), little is known about the endogenous pacemaker processes that control the biology of symbiotic organisms. To date, only one study has used an aposymbiotic cnidarian, *Exaiptasia diaphana*, for studying temporal patterns of behavior and gene expression to understand better the regulation of the host’s clock in the absence of the photosymbiont (*19*). However, while this widely distributed symbiotic sea anemone has become a popular model for coral research, because of key ecological differences between *E. diaphana* with reef building corals (*20*) the degree to which there is cross-talk between host and symbiont clock mechanisms in other symbiotic cnidarians remains unclear. Thus, the coral-Symbiodiniaceae symbiosis makes an attractive system for understanding the integration between the biological clocks of two eukaryotes living together.

This study aimed to investigate natural rhythms in corals and the effect of symbiosis on biological rhythms using the calcifying symbiotic coral *Euphyllia paradivisa* as a model. *E. paradivisa* is highly abundant in the mesophotic reef (36 to 72 m) of the Gulf of Eilat (Aqaba), Red Sea, and was shown to exhibit high physiological plasticity (e.g., tolerance to high irradiance and bleaching conditions, high competitive abilities, successful symbiont adaptation, and survival without algal symbionts during prolonged darkness) (*21*). Despite the association with Symbiodiniaceae from the genera *Cladocopium* (formerly clade C), *E. paradivisa* has already provided proof of its utility as a model for understanding different aspects of symbiotic complexity due to the ability to generate viable aposymbiotic morphs (*21*–*23*).

Here, we applied comparative transcriptomic methods to examine temporal patterns of gene expression in the coral host using symbiotic and aposymbiotic morphs, sampled over 48 hours under natural light/dark (LD) cycles and constant darkness (DD). The rhythmic transcriptomic analysis uncovered 24- and 12-hour periods of gene expression under both light conditions, yet the diel patterns were predominant in both *E. paradivisa* morphs. In addition, we found that in both morphs, the 24-hour cycling transcripts cluster into four groups that differ in their time of peak and trough expression, while the 12-hour transcripts cluster into two groups. Together, these clusters demonstrate the broad rhythmic transcriptomic expression spectrum of *E. paradivisa*. We identified a substantial number of transcripts (*n* = 347; ~4.5% from both symbiotic and aposymbiotic morphs) oscillating with a 24-hour period under both LD and DD, including genes likely involved in the circadian clock of other cnidarians, indicating that an endogenous circadian clock exists in *E. paradivisa*. Our results also revealed various transcripts that display either a 24- or a 12-hour period depending on the corals’ symbiotic state, suggesting that the presence of the photosynthetic algae is associated with changes in rhythmic gene expression.

## RESULTS

### Transcriptome rhythmic analysis

**Fig. 1.**
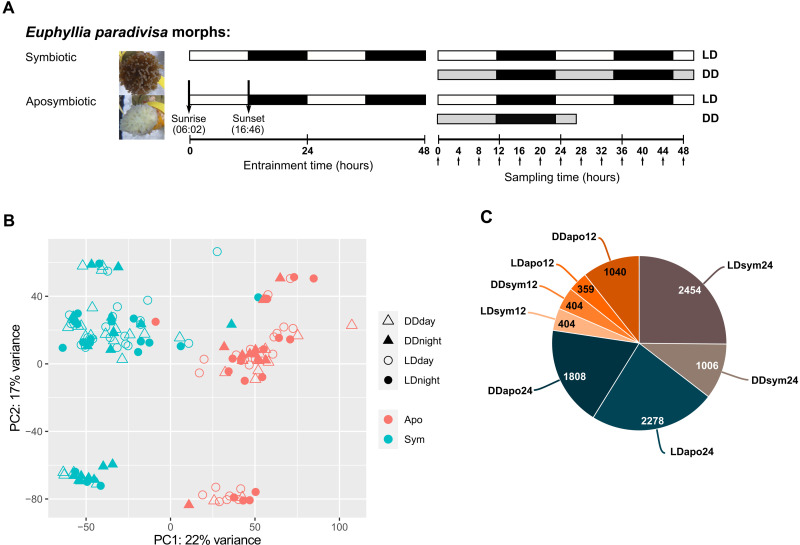
Transcriptome rhythmic analysis. (**A**) Schematic showing the experimental design. Symbiotic and aposymbiotic *E. paradivisa* polyps were sampled under natural LD or DD. Horizontal boxes represent the light and dark phases during entrainment and sampling. White boxes indicate light, black boxes indicate dark, and gray boxes indicate dark during subjective day. Arrows (pointing up) indicate sampling time points. Time 0 = sunrise and time 12 = 1 hour after sunset. (**B**) Principal components analysis (PCA) plot showing the normalized transcriptomic data from symbiotic and aposymbiotic samples (*n* = 138). (**C**) Classification of the 9753 transcripts obtained from rhythmicity analysis using RAIN algorithm (*P* < 0.01) into eight subgroups based on their oscillation period (i.e., 24- or 12-hour period) and experimental subgroup (i.e., symbiotic or aposymbiotic morphs under LD or DD). The number of rhythmic transcripts found in each subgroup and the referred name of the group are reported on the pie chart.

To study the natural rhythms and the symbiosis effect on rhythmic gene expression, we assembled a de novo transcriptome for the coral *E. paradivisa* using symbiotic and aposymbiotic polyps collected at 4-hour intervals under natural LD cycles or DD (Fig. 1A). To generate the aposymbiotic morph, *E. paradivisa* polyps were kept in total darkness for a year. Each polyp had a biological replicate (fragments from the same colony) kept under ambient LD cycles. After the 12-month dark period, *E. paradivisa* polyps lost their original color and became white (Fig. 1A). The de novo transcriptome comprised 115,303 transcripts with an N50 of 693 and a median contig length of 327 and contained 54,053 open reading frame (ORF) encoding transcripts. We were able to assign annotation to 27,080 transcripts from the newly identified transcriptome. A principal components analysis (PCA) of the entire normalized dataset showed that the symbiotic and aposymbiotic samples are segregated into two main groups (PC1, 22% variance), demonstrating that the symbiotic state of the coral has a more substantial effect on gene expression than the light condition (Fig. 1B). PC2 (17% variance), which quite similarly influences gene expression, represents an unknown factor that separates the samples into two smaller distinguished groups. As all known designed factors (light treatment, light phase, sampling time, and sample name) were analyzed, we postulate that it could represent genetic variation.

We used the RAIN (rhythmicity analysis incorporating non-parametric methods) algorithm (*24*) to detect the putative cycling transcripts in the *E. paradivisa* transcriptome. In total, we found that 9753 transcripts (i.e., 8.4% of the transcriptome) show either a 24- or a 12-hour oscillation period (Benjamini-Hochberg–corrected RAIN *P* values < 0.01); 43.6% of the 24-hour period transcripts and 32.2% of the 12-hour period transcripts were annotated. The significant cycling transcripts were classified into eight rhythmic subgroups according to their oscillation period and experimental subgroup, referred to here as LDsym24 (24-hour period in symbionts under LD), DDsym24 (24-hour period in symbionts under DD), LDsym12 (12-hour period in symbionts under LD), DDsym12 (12-hour period in symbionts under DD), LDapo24 (24-hour period in aposymbionts under LD), DDapo24 (24-hour period in aposymbionts under DD), LDapo12 (12-hour period in aposymbionts under LD), and DDapo12 (12-hour period in aposymbionts under DD). The lists of rhythmic transcripts are provided in data file S1. The number of oscillating transcripts differed between these subgroups, though, in both morphs, most genes cycled with a 24-hour period (Fig. 1C). A subsequent Venn diagram analysis showed that ~16% (*n* = 1518) of the cycling transcripts were included in more than one rhythmic subgroup (data file S1I).

### Diel gene expression

**Fig. 2.**
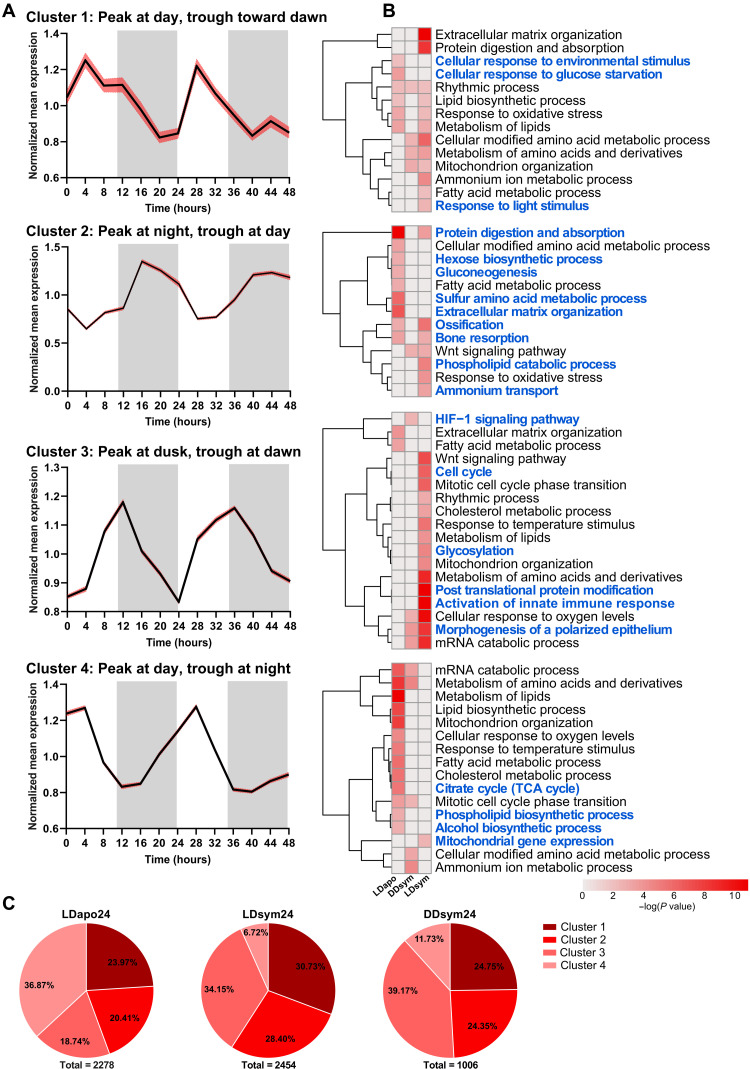
Diel rhythmic gene clustering. Gaussian mixture models (GMMs) clustering generated four diel gene clusters among the LDapo24, LDsym24, and DDsym24 subgroups. (**A**) Mean expression profiles (black lines) with 95% confidence interval (red shading) for each cluster over 48 hours. The profiles’ peak (maximum) and trough (minimum) expression characterize each cluster. White and gray shaded areas indicate the LD phases during the experimental time course. White indicates light or dark during subjective day and gray indicates dark. (**B**) Heatmaps showing significantly enriched ontology terms [GO biological processes, Kyoto Encyclopedia of Genes and Genomes (KEGG) pathway, Reactome gene sets, canonical pathways, CORUM (comprehensive resource of mammalian protein complexes), and WikiPathways] identified by Metascape. Each heatmap represents enriched terms across the three subgroup gene lists in a single cluster (top to bottom: clusters 1, 2, 3, and 4). Color scale represents −log_10_(*P*). Gray cells indicate the lack of enrichment for that term in the corresponding gene list. Blue text indicates terms found enriched in a single cluster. (**C**) Pie charts showing the distribution (percentages) of the rhythmic transcripts per cluster in each subgroup.

To find the broad diel transcriptomic expression pattern of *E. paradivisa*, we applied Gaussian mixture model (GMM) clustering using the rhythmic transcripts (RAIN *P* < 0.01) from LDsym24, DDsym24, and LDapo24. Of note, the DDapo subgroup was excluded from this analysis as they were only sampled during the first 24 hours of the experiment due to the limited viable aposymbiotic polyps available. The analysis revealed four gene clusters that we characterized using the peak and trough time points (i.e., when the normalized expression level is highest and lowest, respectively) of each cluster’s mean profile. The mean expression profiles of clusters 1 and 4 show a peak at daytime and trough during the night; however, in cluster 4, the trough is early at night, while in cluster 1, the trough is toward dawn. The opposite trend can be seen in cluster 2, where the peak is at night and the trough is at daytime. In cluster 3, the mean profile displays a peak at dusk and a trough at dawn (Fig. 2A). Clusters 1 and 4 comprise ~61% (*n* = 1386 of 2278) of aposymbiotic diel rhythmic transcripts, whereas 36.8% (*n* = 840 transcripts) are included in cluster 4. In contrast, for the symbiotic rhythmic transcripts, cluster 4 encompasses the smallest portion [6.72% (*n* = 165 of 2454) and 11.73% (*n* = 118 out of 1006) from LDsym24 and DDsym24, respectively], while 34.15% (*n* = 838 transcripts from LDsym24) and 39.17% (*n* = 394 transcripts from DDsym24) are included in cluster 3. Nevertheless, only 18.74% (*n* = 427) of the aposymbiotic rhythmic transcripts are included in cluster 3. In addition, for all three subgroups, cluster 1 comprises the second largest portion of rhythmic transcripts (23.97, 30.73, and 24.75% from LDapo24, LDsym24, and DDsym24, respectively); however, for DDsym24, the portion of transcripts is similar to that in cluster 2 (Fig. 2C and data file S2A).

We performed functional enrichment analyses over the four clusters using Metascape (*25*) to identify biological processes and pathways carried out by the rhythmic genes of each *E. paradivisa* subgroup at different times of the day. We found that 40 to 65% of the significantly enriched ontology terms were exclusive to a single cluster. Furthermore, among each cluster, most of these terms were uniquely enriched for only one of the *E. paradivisa* subgroups. This demonstrates a time-based segregation between processes that differs between the symbiotic and aposymbiotic morphs (Fig. 2B). For the LDapo24 subgroup, biological processes and pathways such as cellular response to glucose starvation and isoprenoid metabolism were uniquely enriched in cluster 1, sulfur amino acid metabolism and hexose biosynthesis were uniquely enriched in cluster 2, and carbohydrate homeostasis was uniquely enriched in cluster 3. In cluster 4, 97% of the exclusive terms were uniquely enriched for the aposymbiotic subgroup, including phospholipid biosynthesis, tricarboxylic acid (TCA) cycle, and alcohol biosynthesis (Fig. 2B and data file S3, A to D). For the symbiotic subgroups, biological processes and pathways such as fatty acid derivative transport and sulfur compound catabolism were uniquely enriched in cluster 1 for LDsym24 and DDsym24, respectively (data file S3A). In cluster 2, phospholipid catabolism and ammonium transport were uniquely enriched for LDsym24 and connective tissue development and circulatory system process for DDsym24 (Fig. 2B and data file S3B). In cluster 3, 76% of the exclusive ontologies were uniquely enriched for the symbiotic morphs, including glycosylation, activation of innate immune response and carbohydrate derivative biosynthesis for LDsym24, and the hypoxia-inducible factor-1 (HIF-1) signaling pathway and inositol lipid–mediated signaling for DDsym24 (Fig. 2B and data file S3C). In contrast, few terms, such as the rhythmic process in cluster 1, developmental processes and cellular component organization or biogenesis in clusters 2 and 3, signal transduction in cluster 3, and transport of small molecules in cluster 4, were found enriched for the three subgroups. However, they were also found enriched in other clusters, yet only for one of the subgroups. Conversely, from the 35 terms enriched for the three subgroups in cluster 3, 15 terms, including noncanonical Wnt signaling pathway and microtubule-based transport, were uniquely enriched in this cluster (Fig. 2B and data file S3, A to D). Regarding other ontologies found enriched in more than one cluster, we identified processes enriched for each of the *E. paradivisa* morphs at different times of the day (i.e., in different clusters). Cellular response to oxygen levels, mRNA catabolism, and ribose phosphate metabolism were enriched in cluster 3 (peak at dusk and trough at dawn) for LDsym24 and DDsym24, while for LDapo24, they were enriched in cluster 4 (peak at day and through early at night). Similarly, cholesterol metabolism, organophosphate biosynthesis, carbohydrate metabolism, cell cycle phase transition, and many immune system processes were enriched in cluster 3 or cluster 4 for LDsym24 or LDapo24, respectively. In addition, some terms that were unique to the symbiotic morphs were enriched at different times for each of the subgroups, e.g., DNA biosynthesis and amine metabolism that were enriched in cluster 3 or cluster 4 for LDsym24 or DDsym24, respectively (Fig. 2B and data file S3, C and D).

**Fig. 3.**
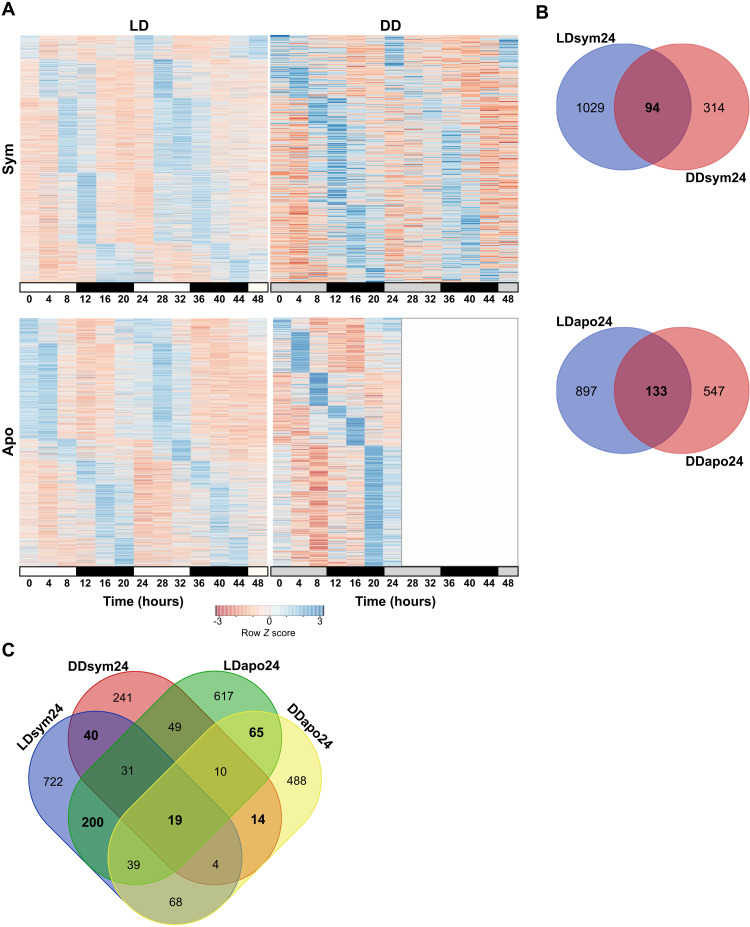
Diel rhythmic gene expression of *E. paradivisa*. (**A**) Heatmaps showing the genes cycling with a 24-hour period (RAIN *P* < 0.01) in symbiotic (top) or aposymbiotic (bottom) morphs under LD (left) or DD (right). Each row represents a gene, and each column represents the median values for a single time point (*n* = 3 biological replicates). Rows are ordered by the estimated phase of oscillation determined by RAIN. Expression level is represented by *Z* score. (**B**) Venn diagrams showing the number of genes cycling under LD and DD in symbiotic (top) or aposymbiotic (bottom) morphs. (**C**) Venn diagram showing the overlap between genes cycling in the LDsym24, DDsym24, LDapo24, and DDapo24 subgroups. Overlapping gene groups resulting from similar morph or similar light treatment are marked in bold.

**Fig. 4.**
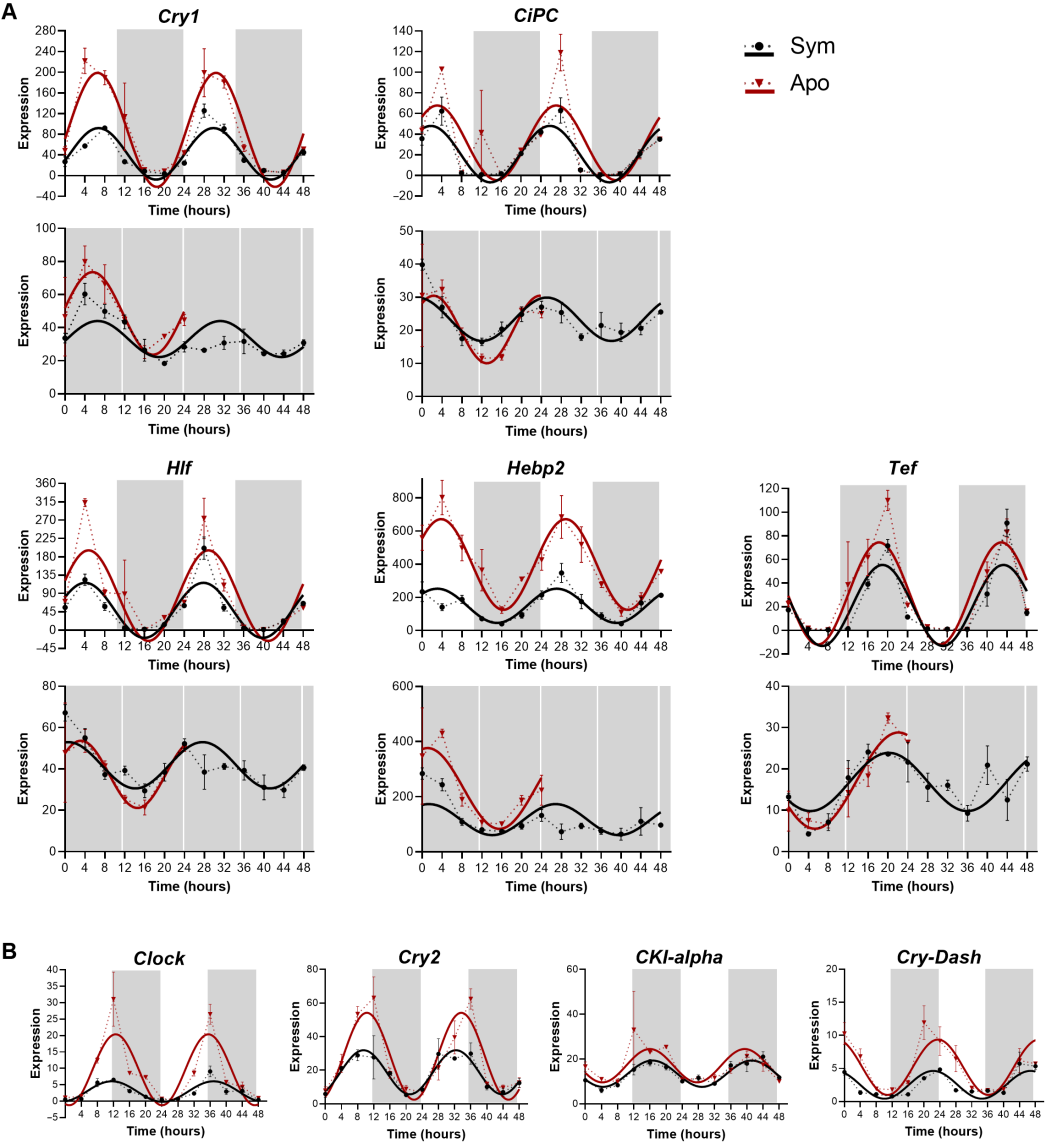
Expression profiles of candidate circadian genes of *E. paradivisa*. (**A** and **B**) Expression plots for candidate core-clock and clock-controlled genes cycling with a 24-hour period (RAIN *P* < 0.01) in symbiotic (black) and aposymbiotic (red) morphs. The *x* axis indicates the experimental time course (time 0 = sunrise and time 12 = 1 hour after sunset). White and gray shaded areas indicate light and dark, respectively. The *y* axis indicates the normalized expression levels. Note that the scales differ across plots. Data points are presented as mean values ± SEM (*n* = 3 biological replicates). Fitting curves (smooth lines) are based on sinusoidal function. (A) Each vertical set of plots represents a single gene’s expression under LD (top) and DD (bottom). (B) Each plot represents a single gene’s expression under LD.

To uncover circadian rhythmicity, we further analyzed the annotated transcripts (hereafter referred genes) found in the four diel rhythmic subgroups (i.e., LDsym24, DDsym24, LDapo24, and DDsym24). We identified 2153 and 1088 genes (including symbiotic and aposymbiotic groups) that oscillated with a 24-hour period under LD and DD, respectively (RAIN *P* < 0.01; Fig. 3 and data file S1, A to D). While in both morphs, most genes cycled only under LD, suggesting that their rhythmicity is light-driven, 6 to 8% of the genes showed oscillatory expression signatures under both LD and DD (*n* = 94 for LDsym24/DDsym24 and *n* = 133 for LDapo24/DDapo24; Fig. 3B). We, therefore, classified these genes as circadian or circadian-controlled genes. Next, we compared the four subgroups and distinguished groups of genes that display circadian or light-dependent expression and are shared between the morphs from those likely influenced by the symbiotic state of the coral (Fig. 3C). The overlapping gene groups included 105 genes displaying oscillatory expression under both LD and DD that is symbiotic state dependent (*n* = 40 in the LDsym24/DDsym24 group; *n* = 65 in the LDapo24/DDapo24 group), which we labeled as “morph-specific” circadian genes. We then explored the distribution of these genes within the four clusters. For the aposymbiotic morphs, 4% of the genes were included in cluster 1, 23% in cluster 2, 35% in cluster 3, and 38% in cluster 4. For the symbiotic morphs, when considering genes from both LDsym24 and DDsym24 that are in the same cluster, we found that 12.5, 7.5, and 38% were included in clusters 1, 2, and 3, respectively, while none of the genes were common to both subgroups in cluster 4. However, also when considering the distribution for each group separately, most genes were included in cluster 3 (*n* = 21 for LDsym24 and *n* = 21 for DDsym24). Among the genes common to the four subgroups, we found the putative core circadian clock components, *Cry1*, *Cipc-like*, *Hlf*, and *Tef*, and one putative antioxidant, heme-binding protein (*Hebp2*), which was reported to follow a synexpression pattern with *Cry1* in *Acropora millepora* (*16*) and *Acropora cervicornis* (*26*) (Figs. 3C and 4A and data file S1I). Of note, since there were four transcripts annotated *Cry1* in our data, we performed an additional phylogenetic evaluation (fig. S1) to bring in line the annotation with other cnidarian species and to examine whether they belong to the novel anthozoan-specific cryptochrome groups described recently by Gornik *et al.* (*27*). Accordingly, the transcript that we refer to in the text as *Cry1* (TRINITY_DN6_c2_g1_i1.p1) cycled under LD and DD in both morphs was the only one classified in the Anthozoan CRY-IIs family, while the remaining, which were rhythmic only under LD, were classified as AnthoCRYs. *Cry1*, *Cipc*, *Hlf*, and *Hebp2* showed similar temporal expression patterns, presenting an expression peak during daytime. In contrast, for *Tef*, the expression peaked late at night (Fig. 4A). Within this small conserved group, two genes encoding E3 ubiquitin-protein ligases, *Trim71* and *Dzip3*, were additionally identified. Known core clock components and clock-related genes of circadian timekeeping found in our data, including *Clock*, *Cry2*, *Casein kinase 1*, and *Cry-DASH*, showed a weak circadian rhythm. While under LD, they were found rhythmic in both morphs (RAIN *P* < 0.01), under DD, the 24-hour period did not uphold the significance threshold (Fig. 4B and table S1).

### Twelve-hour rhythms of gene expression

**Fig. 5.**
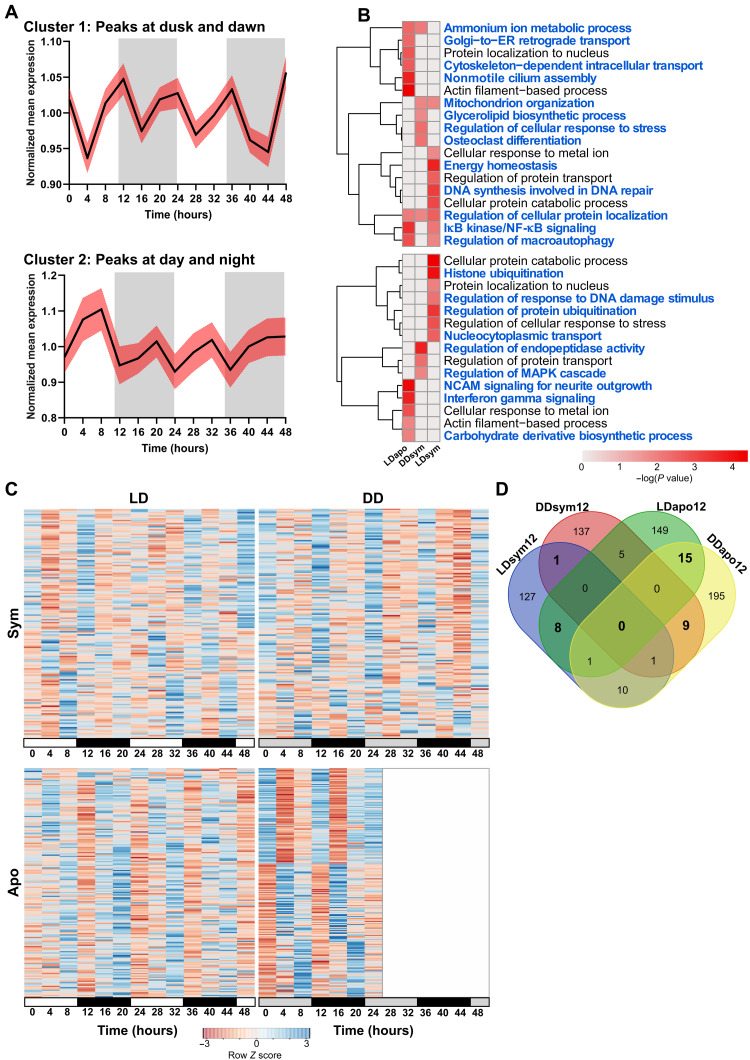
Tidal rhythmic gene expression of *E. paradivisa*. GMM clustering generated two gene clusters among the LDsym12, DDsym12, and LDapo12 subgroups. (**A**) Mean expression profiles (black lines) with 95% confidence interval (red shading) for each cluster over 48 hours. The profiles’ expression peaks (maximum) characterize each cluster. White and gray shaded areas indicate the LD phases during the experimental time course. White indicates light or dark during subjective day and gray indicates dark. (**B**) Heatmaps showing significantly enriched ontology terms (GO biological processes, KEGG pathway, Reactome gene sets, canonical pathways, CORUM, and WikiPathways) identified by Metascape. Each heatmap represents enriched terms across the three subgroup gene lists in a single cluster. Color scale represents −log_10_(*P*). Gray cells indicate the lack of enrichment for that term in the corresponding gene list. Blue text indicates terms found enriched in a single cluster. (**C**) Heatmaps showing the genes cycling with a 12-hour period (RAIN *P* < 0.01) in symbiotic (top) or aposymbiotic (bottom) morphs under LD (left) or DD (right). Each row represents a gene, and each column represents the median values for a single time point (*n* = 3 biological replicates). Rows are ordered by the estimated phase of oscillation determined by RAIN. Expression level is represented by *Z* score. (**D**) Venn diagram showing the overlap between genes cycling in the LDsym12, DDsym12, LDapo12, and DDapo12 subgroups. Overlapping gene groups resulting from similar morph or similar light treatment are marked in bold.

To explore the 12-hour transcriptomic rhythm, resembling tidal pace, of *E. paradivisa*, GMM clustering was applied using the 1167 rhythmic transcripts from the LDsym12, DDsym12, and LDapo12 subgroups. The analysis revealed two clusters with opposite expression profiles (Fig. 5A and data file S2). The mean expression profile of cluster 1 demonstrates two peaks occurring at dusk and dawn, while the troughs are at day- and nighttime. In contrast, the mean profile of cluster 2 shows that the peaks occur at day- and nighttime, whereas the troughs are at dusk and dawn.

The functional enrichment analysis revealed that 95% of the significant ontology terms were uniquely enriched in each cluster and only for one of the *E. paradivisa* subgroups (Fig. 5B and data file S3, E and F). In cluster 1, processes and pathways such as nonmotile cilium assembly, response to interleukin-1, and Golgi–to–endoplasmic reticulum (ER) retrograde transport were found uniquely enriched for LDapo12; DNA synthesis involved in DNA repair and multicellular organismal homeostasis were found uniquely enriched for LDsym12; and leukotriene metabolism, glycerolipid biosynthesis, and osteoclast differentiation were found uniquely enriched for the DDsym12 subgroup. In addition, few of the unique terms were found enriched for two subgroups. These include mitochondrion organization, enriched in LDsym12 and DDsym12; inhibitor of nuclear factor κB (NF-κB) kinase/NF-κB signaling, enriched in LDsym12 and LDapo12; ammonium ion metabolism, enriched in LDapo12 and DDsym12; and a single process, regulation of cellular protein localization, which was found enriched for all three subgroups (Fig. 5B and data file S3E). In cluster 2, all the ontology terms were found uniquely enriched for a single subgroup. For example, interferon-γ signaling and carbohydrate derivative biosynthesis were uniquely enriched in LDapo12; histone ubiquitination, intracellular steroid hormone receptor signaling pathway, heterocycle catabolism, and nuclear transport were enriched in LDsym12; and regulation of peptidase activity was enriched in the DDsym12 subgroup (Fig. 5B and data file S3F). Among the ontology terms found enriched in both clusters, few showed enrichment for each morph in a different cluster (i.e., enriched in cluster 1 for LDapo12 and in cluster 2 for LDsym12 or vice versa), including protein localization to nucleus and cellular response to metal ion. In addition, five terms (e.g., regulation of protein transport and organic hydroxy compound metabolism) were unique to the symbiotic morphs; however, each of the subgroups showed enrichment in a different cluster (i.e., enriched in cluster 1 for LDsym12 and cluster 2 for DDsym12 or vice versa; Fig. 5B).

In seeking further endogenous tidal pace, we focused on the annotated transcripts, i.e., genes. In total, 326 and 384 genes oscillated with a 12-hour period under LD and DD, respectively (RAIN *P* < 0.01; Fig. 5C and data file S1, E to H). By comparing the four 12-hour subgroups (Fig. 5D), we found for each morph different genes that cycled under both LD and DD and, therefore, are possibly regulated internally and influenced by the symbiotic state of the coral. These include only 1 gene in the symbiotic morphs, γ-glutamylamine-cyclotransferase (*Ggact*), which possibly functions in the latter catabolism stages of the products of transglutaminases, a reaction resulting in the formation of ammonia (*28*), and 15 genes, including *Cpt1a* (carnitine palmitoyltransferase 1A), *Map3k7* (mitogen-activated protein kinase kinase 7), *Traf6* (tumor necrosis factor receptor–associated factor 6), *Trim3* (tripartite motif–containing 3), *Trim71* (tripartite motif–containing 71), *Stip1* (stress-induced phosphoprotein 1), *Pgbd1* (piggyback transposable element–derived 1), and *Fah* (fumarylacetoacetate) in the aposymbiotic morphs. These genes are broadly implicated in stress and immune responses (*29*–*34*). In addition, two gene groups were marked likely light-driven, as in both morphs, they oscillate either under LD or under DD. Among these, eight genes (including *Cacna2d1*, *Rnf213*, *Fibcd1*, *Prkn*, and *Macf1*) were found in LDsym12 and LDapo12, and nine genes (including *Gbp4*, *Csnk2b*, *Traf3*, *Tnfrsf22*, and *Cd109*) were found in DDsym12 and DDapo12, with the latter also including many genes involved in immune regulation [such as *Traf3* (*35*), *Tnfrsf22* (*36*), and *Gbp4* (*37*)].

### Twenty-four-hour to 12-hour period alternations in gene expression

**Fig. 6.**
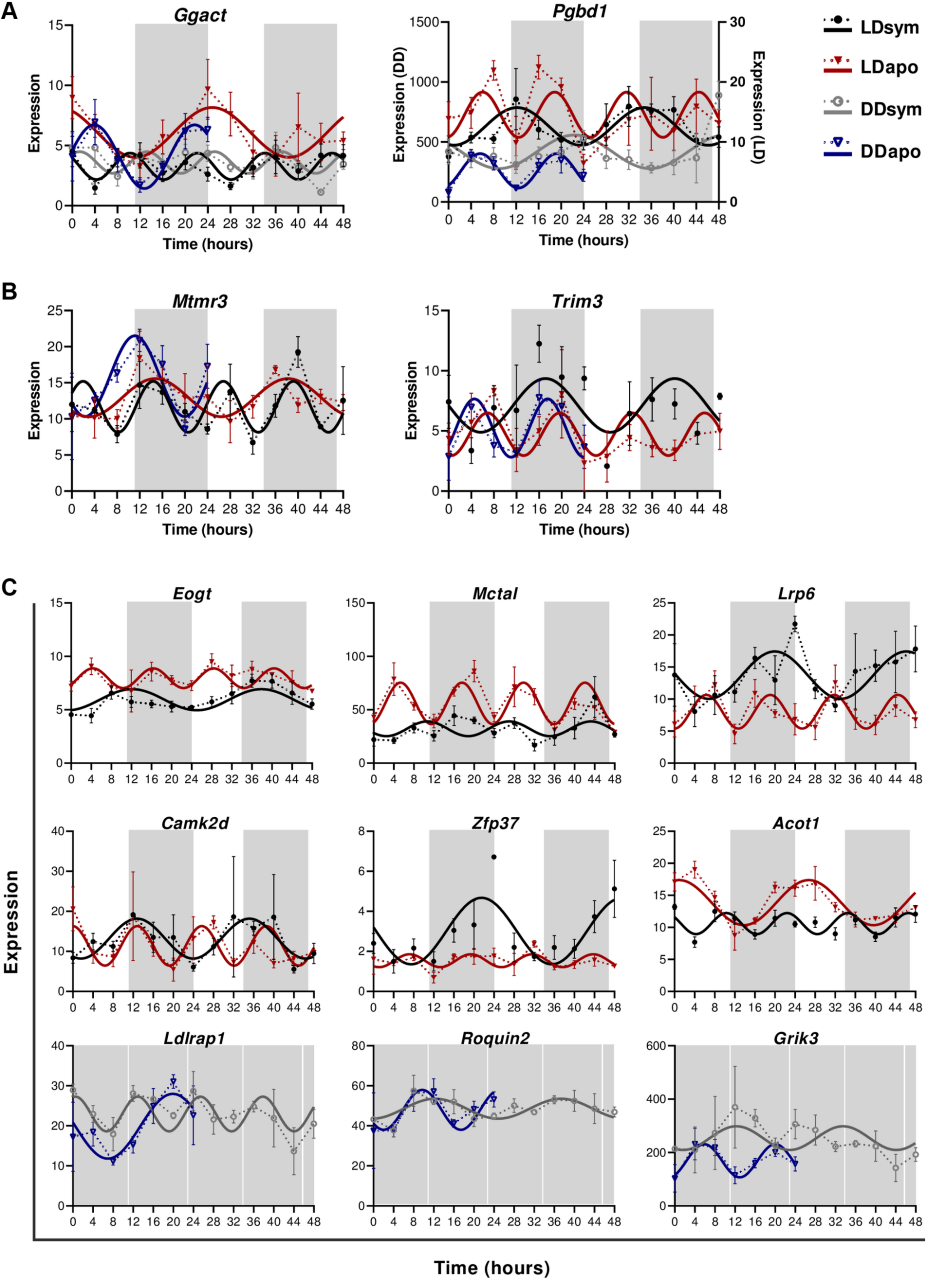
Twenty-four-hour to 12-hour period alternations among symbiotic and aposymbiotic *E. paradivisa*. Expression plots for selected genes cycling with either a 24-hour or 12-hour period (RAIN *P* < 0.01) under (**A**) LD and DD in symbiotic (black and gray) and aposymbiotic (red and blue) morphs, (**B**) only LD or LD and DD in symbiotic or aposymbiotic morphs, respectively, and (**C**) only LD or only DD in both morphs. The *x* axis indicates the experimental time course (time 0 = sunrise and time 12 = 1 hour after sunset). The *y* axis indicates the normalized expression levels. Note that the scales differ across plots. Data points are presented as mean values ± SEM (*n* = 3 biological replicates). Fitting curves (smooth lines) are based on sinusoidal function. White and gray shaded areas indicate the LD phases during the experimental time course. White indicates light or dark during subjective day and gray indicates dark.

By comparing the eight rhythmic subgroups, we uncovered 59 genes (of 114 transcripts) that showed altered periodicity, from 24 to 12 hours (or vice versa), apparently due to the corals’ symbiotic state. (Fig. 6 and table S2). Two genes showed oscillatory expression signatures under both LD and DD in each morph yet cycled with either a 24- or a 12-hour period. *Ggact* oscillated with a 24-hour period in aposymbiotic morphs and a 12-hour period in symbiotic morphs, while *Pgbd1* cycled with a 24-hour period in symbiotic morphs and a 12-hour period in the aposymbionts (Fig. 6A). Similarly, listed genes that demonstrated a period alteration but oscillated under both LD and DD only in aposymbionts included *Trim3*, found in LDsym24, LDapo12, and DDapo12, and *Mtmr3* (myotubularin-related protein 3), found in LDsym12, LDapo24, and DDapo24 (Fig. 6B). Furthermore, we distinguished four groups of genes that displayed a period alternation only under one of the light conditions. Two groups include genes that cycled with a 24-hour period in symbiotic morphs and a 12-hour period in aposymbiotic morphs, such as *Eogt*, *Slc25a29*, *Lrp6*, and *Camk2d* that were rhythmic under LD, and *Grik3*, *Roquin2*, *Sf1*, and *Cnot3* under DD. Genes in these two groups are enriched in housekeeping functions of gene regulation (data file S3G). The other two groups demonstrated the opposite trend, i.e., a 24-hour period in aposymbionts and a 12-hour period in the symbionts, including *Acot1*, *Ankmy2*, and *Lrp4* that were rhythmic under LD, and *Ldlrap1*, *Cers1*, and *Fgf6* under DD (Fig. 6C). While no gene ontology (GO) terms are revealed with a *P* value smaller than 0.01 in these genes, glucose and lipid metabolism pathways are nevertheless weakly enriched (data file S3H).

## DISCUSSION

Our transcriptomic rhythmic analysis comparing symbiotic and aposymbiotic coral morphs, subjected to natural LD cycles or DD, revealed that diurnal and tidal gene expression patterns exist in the coral *E. paradivisa*, irrespective of the presence of Symbiodiniaceae. Moreover, it was found that although the cycling transcripts mainly differed between the symbiotic and aposymbiotic corals, they composed six clusters, all of which include transcripts from both morphs. The clusters, which illustrate the broad rhythmic transcriptomic expression spectrum of *E. paradivisa* throughout the day, exposed variations between the two morphs (Figs. 2 and 5). These variations point out the complex regulatory mechanisms in the coral-algal symbiosis and suggest that the host’s rhythmic expression is strongly reliant on the symbiotic state. This is strongly evident in the four diel clusters, particularly in cluster 3 and cluster 4.

**Fig. 7.**
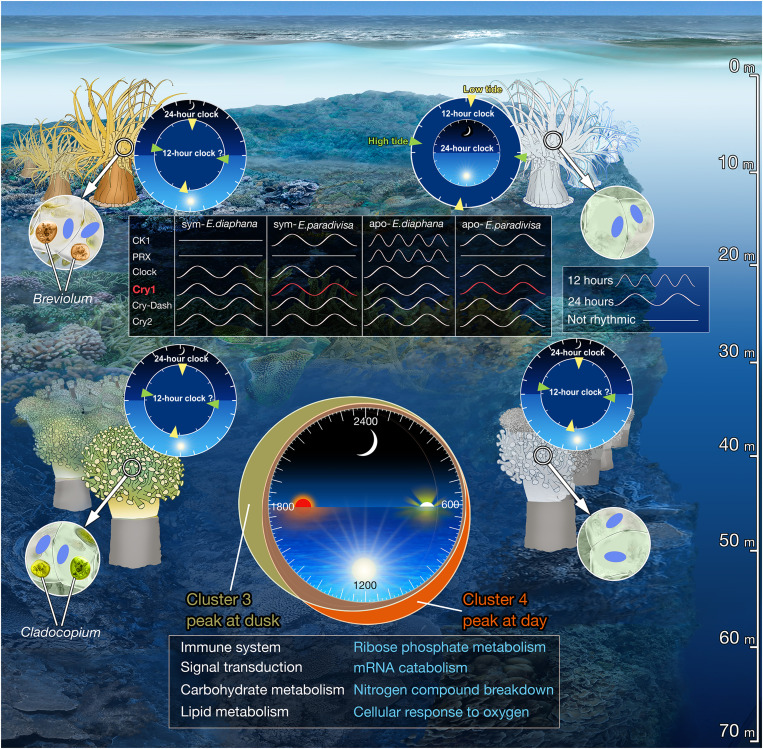
Conceptual summary comparing *E. paradivisa* and *E. diaphana*. Illustration of main finding in symbiotic (left) and aposymbiotic (right) *E. diaphana* (top) and *E. paradivisa* (bottom). The four clocks indicate the periodicity of rhythmic gene expression with the predominant period mentioned in the outer ring. The periodicity of putative circatidal and circadian genes is indicated in the top box. Red indicates oscillatory expression under LD and DD. Rhythmic processes enriched at dusk or daytime for symbiotic or aposymbiotic corals, respectively, are mentioned in the bottom box. Blue text indicates processes that are under circadian regulation.

In unicellular algae and free-living dinoflagellates, the daily LD cycles are tightly coupled to the circadian clock, which regulates various processes such as photosynthetic oxygen evolution, carbon fixation, nutrient acquisition, cell-cycle progression, and lipid biosynthesis (*38*–*41*). Microarray analysis of *Symbiodinium* cultured under both LD and LL conditions showed that 30.1% of the genes targeted on the array displayed diel oscillations under LD conditions, and 7.1% of these genes were suggested to be under circadian regulation (*42*). However, only photosynthesis has been shown to be coupled to the endogenous circadian clock in both the free-living and in hospite forms (*43*). De Los Reyes *et al.* (*44*) suggested that evolution has mainly conserved rhythmic genes with expression patterns that are strongly influenced by the dark-to-light transition at dawn and light-to-dark transition at dusk in photosynthetic organisms. Accordingly, our results may indicate that the host’s rhythmic expression, in its symbiotic state, could be tightly coupled to the symbionts’ diel expression, and consequently, many transcripts peak and trough during the light transitions. Moreover, our data show that many rhythmic processes in the coral’s biology, including cellular component organization or biogenesis, immune system processes, signal transduction, and several metabolic processes, are conserved between the morphs. However, in the symbiotic corals, most processes are strongly enriched during the light transition at dusk (i.e., cluster 3), while for the aposymbionts, they are mainly enriched during daytime (cluster 4), suggesting that the symbiotic algae modify the timing of the host’s rhythmic processes along the day (Fig. 7).

Consistent with previous analyses of other cnidarian transcriptomes (*45*–*47*), our results revealed that most of the 24-hour cycling transcripts of *E. paradivisa* oscillate only under LD, yet ~4.5% maintain a similar oscillation period under DD. The persistence of diel oscillatory expression under DD, particularly in the aposymbiotic morphs, manifestly indicates that an endogenous circadian oscillator exists in *E. paradivisa*. The bulk of these circadian transcripts was unique to only one of the morphs, implying that, as for most diurnally expressed transcripts, the circadian regulation of the coral host is strongly dependent on its symbiotic state. The distribution of these morph-specific circadian genes throughout the day further emphasizes this dependency. While in the symbiotic corals, circadian genes peaking at dusk are the most abundant, the aposymbiotic genes demonstrate a more diffuse distribution (i.e., peaking at day, dusk, and night). These findings could suggest that Symbiodiniaceae alters the primary organization of the host’s circadian rhythm that is somewhat independent of the light transitions.

Several studies have demonstrated that changes in metabolite, nutrient, and redox state, imposed by the endosymbiotic algae, occur within the host’s tissue during the daily LD cycles. These changes, mainly driven by the circadian photosynthetic rhythm, were suggested to influence cnidarians’ rhythmicity (*42*, *48*–*52*). We postulate that the corals use several molecular mechanisms to incorporate diverse signals along with LD cycles and regulate rhythmic expression over their transcriptome. In this view, our diel rhythmic data present the potential integration of external (i.e., light) and internal (i.e., redox and metabolic state of the tissue) cues used by symbiotic cnidarians to regulate diel rhythmicity, either in entraining their circadian oscillator or as a direct response. We hypothesize that aside from the direct response, light acts as the primary cue in entraining the endogenous oscillator of the aposymbiotic corals. On the other hand, in symbiotic *E. paradivisa*, light primarily entrains the Symbiodiniaceae clock, which drives internal temporal changes that act as the predominant cues in synchronizing the host’s oscillator. Coordinating these two independent clocks perhaps allows the holobiont to anticipate the daily light transitions and prepare for the upcoming environmental and internal changes.

Our diel enrichment analysis indicates that in symbiotic *E. paradivisa*, biological processes and pathways, including cellular response to oxygen levels, nitrogen compound breakdown, mRNA catabolism, and ribose phosphate metabolism, are under circadian regulation. Although our analyses could not uncover whether these processes, as well as all other enriched ontologies, are under circadian regulation in the aposymbiotic morphs, they were found enriched. However, symbiotic corals showed enrichment at dusk and aposymbiotints at daytime (Figs. 2B and 7). These findings could suggest that the host’s circadian-regulated processes, like many other diel processes, are synchronized to follow the symbiont’s rhythm to maintain the mutualistic relationship in an advantageous manner. While further research is needed to determine the mechanisms by which the host and symbiont sense the daily environmental and internal changes and if/how they use these changes to synchronize each other’s clocks, our data likely support the hypothesis that the symbiotic algae control the host’s rhythmicity (*16*, *19*).

An important finding in our data is the precise diel rhythm of the candidate circadian clock genes, previously reported rhythmic for other cnidarians (*19*, *45*, *46*, *53*, *54*), in both *E. paradivisa* morphs (Fig. 4). Some of these genes, such as *Cry1* and *Cipc-like*, maintained a consistent 24-hour period under LD and DD, indicating valid circadian regulation. In contrast, others, including *Clock* and *Cry2*, showed a weak circadian rhythm, suggesting that they could be involved in light-dependent circadian regulation. These results support the findings in *E. diaphana* (*19*) and further indicate that in cnidarians, the diel rhythmicity of circadian clock components is not influenced by the presence of their symbionts (Fig. 7).

Several studies have shown that in cnidarians, cryptochrome genes display diel oscillatory expression that peaks at daytime under persistent LD cycles (*19*, *26*, *47*, *55*). Moreover, it was suggested that they act as photoreceptors that can mediate environmental light signals in entraining the circadian oscillator (*56*). Recently, Gornik *et al.* (*27*) identified two distinct Cry groups in the Anthoza, namely, Anthozoan-CRY-IIs and AnthoCRYs. On the basis of their phylogenetic analysis, we classified one transcript in the Anthozoan-CRY-IIs group (i.e., *Cry1*), four AnthoCRYs (including the transcript assigned *Cry2*), and one Cry-Dash (fig. S1). Moreover, although they speculate that Anthozoan-CRY-IIs are involved in the circadian clock function, they found that in *E. diaphana*, the rhythmic expression of AnthoCry and CRY-II is light-driven (*27*). In *E. paradivisa*, under LD, the expression of *Cry1* peaks earlier in the day than *Cry2*, which peaks toward dusk, and *Cry-DASH* peaks at dawn. However, one outstanding discovery is the rhythmic expression of *Cry1* that is maintained under DD. Corals living in the deeper reef are mainly exposed to low blue light intensity (*57*), and thus, these three cryptochromes may act differently in signaling the circadian clock in *E. paradivisa*, which inhabits the mesophotic reef (below 40 m), as opposed to the symbiotic corals and anemones living in shallow water (Fig. 7).

In mammals, *Cry1* and *Cry2* act as light-independent inhibitors of the CLOCK-BMAL1 heterodimer (*58*) and are essential for maintaining circadian rhythmicity (*59*). Because of the loss of rhythmicity of *Cry* genes in other cnidarians (*46*, *56*, *60*), it was proposed that, as in *Drosophila* (*61*), in the negative feedback loop of the cnidarian molecular clock, CRY binds to TIMELESS2/TIMEOUT in a light-dependent manner to serve as a repressor of transcription (*55*, *62*). Our results could suggest that in the coral *E. paradivisa*, *Cry1* acts as a light-independent repressor in the negative feedback loop, while *Cry2* and *Cry-DASH* act in entraining the oscillator during the LD transitions.

It is also important to mention that in our rhythmic data, two of the *Cry1* homologs showed a 24-hour period only under LD regardless of the coral’s symbiotic state. However, in the symbiotic morphs, one of these transcripts showed both 24- and 12-hour periods, probably due to additional peaks at dusk. In *Drosophila* and mammalians, the activity of CRY proteins depends on the cellular redox state (*63*, *64*). Therefore, our results can suggest that in *E. paradivisa*, the CRY proteins similarly act as both components of the TTFL and as sensors of various factors. Moreover, the coexpression of *Cry1* and *Hebp2* supports the hypothesis of an ancestral trait linking circadian clocks to the cellular redox system, and *Cry* potentially represents one such link (*16*, *26*, *51*, *65*).

In the last decade, few studies have provided molecular evidence for an endogenous circatidal oscillator, with an approximately 12.4-hour period, which is distinct from the circadian clock in coastal and estuarine animals (*9*, *12*, *66*, *67*). Recently, it was found that, as in marine animals, the mammalian 12-hour rhythms are governed by a cell-autonomous 12-hour clock that mediates diverse biological pathways (*68*). The finding of several 12-hour rhythmic genes that are conserved in different species indicates potential evolutionary conservation of a circatidal clock (*10*).

In contrast to the finding of predominant diel oscillations in both *E. paradivisa* morphs, a dominant tidal rhythm was found in aposymbiotic *E. diaphana* studied under LD cycles (*19*). In our rhythmic data, conserved circatidal markers, including *Casein kinase 1*, *Peroxiredoxin*, *Xbp1*, and mitochondria DNA–encoded genes, either oscillated with a 24-hour period or were absent (Fig. 7). In addition, ∼5% of the 12-hour period transcripts overlap between the symbiotic and aposymbiotic subgroups, and only a few genes (e.g., *Ggact*, *Map3k7*, *Cpt1a*, *Stip1*, *Trim3*, and *Traf6*) within each morph oscillated under both LD and DD. Many of these 12-hour genes are involved in stress and immune response in aposymbiotic morphs. Since emerging evidence indicates that the immune signals are integral components of the expanded cell-nonautonomous proteostasis network that activate protein quality control mechanisms in response to various stresses (*69*), this may reflect an adaptive response of *E. paradivisa* to 12-hour fluctuating environmental changes associated with tidal cues. These findings could further indicate that exogenous cues, other than light, entrain the oscillator to the tidal cycles, which may be rationalized considering *E. paradivisa* exposure to low light intensity in its natural habitat. However, given the overall low abundance of transcripts expressed in a tidal fashion and the lack of molecular markers, the presence of an independent circatidal clock in *E. paradivisa* remains questionable. It seems reasonable to suggest that our data support the mammalian harmonics theory, according to which the noncompetitive binding of two circadian transcription activators or repressors oscillating in antiphase is capable of driving 12-hour rhythms in gene expression in a cell-autonomous manner (*70*, *71*).

Despite the lack of circatidal markers, eight genes following tidal rhythmicity in *E. paradivisa* morphs are found oscillating with a 12-hour period in mouse liver (*68*). One of these genes, which encodes a lipid phosphatase (*Mtmr3*), altered from a 24-hour oscillation period in aposymbionts to a 12-hour period in the symbiotic morphs, while the others, including an ER-localized transferase (*Eogt*) and a mitochondria transporter (*Slc25a29*), altered from a 24-hour period in symbiotic morphs to a 12-hour period in the aposymbionts. It was suggested that the 12-hour cell-autonomous clock found in mice has an ancient origin due to the conserved 12-hour expression of ER stress and mitochondria homeostasis genes in mice with those found in nematodes and crustaceans (*12*, *68*). Finding such genes with a 12-hour oscillation period only in aposymbiotic corals could indicate an even more ancient role of 12-hour rhythms that are conserved throughout evolution. Moreover, biological pathways with tidal rhythms enriched in *E. paradivisa* morphs are also identified with 12-hour rhythms in mouse livers, including NF-κB signaling, protein transport, proteolysis, translation initiation, ubiquitin-dependent protein degradation, pre-mRNA processing, and *N*-linked glycosylation (*10*, *68*). These data suggest that while individual genes displaying ~12-hour rhythms may not be strongly conserved for different species, the underlying biological functions of tidal rhythms, especially those regulating genetic information transfer (i.e., genes that are involved in basal transcription, mRNA processing, translation, and protein processing) and immune regulation, are maintained during evolution (*72*).

Periodicity alternations, which are symbiotic-state dependent, have also been demonstrated in *E. diaphana* (*19*). It was shown that the genes shifting from a 24-hour period in symbiotic morphs to a 12-hour period in the aposymbionts, which were more abundant than those altering in the other direction, were highly enriched with pathways involved in the regulation of translation in response to nutrition or stress (*19*). While our data show similar portions of transcripts altering in either direction between symbiotic and aposymbiotic *E. paradivisa*, genes shifting from a 24-hour period in symbiotic morphs to a 12-hour period in the aposymbionts are also involved in gene regulation, although in this case, they are more involved in mRNA metabolism at the posttranscriptional step. These results suggest the 12-hour tidal rhythms of genetic information flow in the cnidarians hosts can be modified and influenced by the presence of Symbiodiniaceae.

Overall, the analysis presented here provides evidence for biorhythmicity in the coral *E. paradivisa* and illustrates the complexity of biological rhythms in symbiotic organisms. At present, biorhythms seem to have large variability within the Cnidaria phylum that could reflect the different habitats and evolutionary plasticity to different environmental rhythms (*55*, *73*). Nonetheless, this work manifests the remarkable impacts Symbiodiniaceae has on the host’s transcriptional rhythms when considering obligatory, rather than facultative, as well as mesophotic, rather than shallow water, symbiotic corals (Fig. 7) (*16*, *19*). Although we are still a long way from uncovering the mechanisms that control the synchrony of the host and symbiont clocks in the coral-Symbiodiniaceae symbiosis, the data demonstrate that the presence of the photosynthetic algae can alter the timing of several metabolic processes, including carbohydrate metabolism, lipid metabolism, and other vital processes and pathways in the coral’s biology. While symbiotic corals primarily acquire fixed carbon from their symbiotic algae, during bleaching and recovery, certain coral species can rely on heterotrophic feeding as their alternative sources of fixed carbon (*74*). The finding of processes linked to carbohydrate and lipid biosynthesis in the aposymbiotic morphs, which likely result from the absence of translocated photosynthates (*75*), supports the hypothesis that corals with symbiont-state plasticity, such as the *Euphyllia* genus, could survive under different climate change scenarios in a bleached state and be capable of replenishing degraded coral reefs in the future (*22*, *74*, *76*, *77*).

## MATERIALS AND METHODS

### Coral collection and experimental design

Ten mature colonies of *E. paradivisa* (measuring 20 to 30 cm) were collected at 40- to 60-m depth from the Gulf of Eilat (Aqaba, Red Sea). The coral collections were made under permit no. 2014/40478 issued by the Israeli Nature and National Parks Protection Authority. The colonies were fragmented into nubbins (measuring ~5 cm) and divided into two groups (total of 200 polyps) to produce symbiotic and aposymbiotic morphs. The two morphs were generated and maintained according to the methods presented in Meron *et al.* (*22*). Before the experiment, photosynthetic efficiency (*F*_v_/*F*_m_) was measured on representative *E. paradivisa* polyps from both groups using a diving PAM (pulse amplitude modulator) fluorometer (Walz). The polyps kept under ambient light (i.e., symbiotic) achieved high photosynthetic efficiency, while in the polyps kept under DD (i.e., aposymbiotic), no fluorescence efficiency was detected (fig. S2). All polyps were then subjected to the natural LD cycle (i.e., sunrise at 06:02 and sunset at 16:46 on 12 November 2016; light intensity of 15 to 52 μmol photons m^−2^ s^−1^ during daytime and seawater temperature of 24°C) during 48 hours for entrainment. Subsequently, the polyps were divided into four experimental subgroups: symbiotic and aposymbiotic morphs held under natural LD or DD. Sampling began at sunrise (i.e., time 0 = 06:03) on 14 November 2016 and was performed at 4-hour intervals over 48 hours. At each time point, four individual polyps were sampled from each experimental subgroup, immediately snap-frozen in liquid nitrogen, and transferred to −80°C for storage. The number of viable aposymbiotic polyps was limited; therefore, under DD, they were sampled only at the first 24 hours of the experiment.

### RNA extraction, library preparation, and sequencing

Total RNA was extracted from all sampled polyps (*n* = 184) using TRIzol reagent (Invitrogen) according to a modified version of the manufacturer’s protocol that includes additional chloroform precipitation and overnight precipitation in 5 M LiCl at −20°C, as described in Rosenberg *et al.* (*78*). Purified RNA samples were analyzed using a NanoDrop 1000 spectrophotometer (Thermo Fisher Scientific) to assess RNA quantity and 2200 TapeStation (Agilent) to assess RNA quality (RNA integrity number, >8.5). From each of the 138 samples (3 biological replicates with the highest-quality extracts × 4 experimental subgroups × 13 time points or 7 time points in the DD aposymbiotic group), 1.5 μg of RNA was sent for library preparation and sequencing at the Technion Genome Center in Haifa, Israel. RNA samples were prepared using the Illumina TruSeq RNA Library Preparation Kit v2, according to the manufacturer’s protocol. Libraries ran on nine lanes of an Illumina HiSeq2500 using the multiplexing strategy of the TruSeq protocol. The protocol starts with polyA selection that results in mRNA selection only. On average, ~16 million paired-end reads, 100 bp long, were obtained for each sample. FastQC (www.bioinformatics.babraham.ac.uk/projects/fastqc/) was used to quality check raw parsed fastq files before further analysis.

### Transcriptome assembly and annotation

De novo transcriptome assembly was based on the paired-end sequences of all 138 samples. To filter out potential Symbiodiniaceae sequence reads, the raw reads were first aligned using BBsplit (https://sourceforge.net/projects/bbmap/) to Symbiodiniaceae sequences that were publicly available at the time of the analysis (November 2018). The following Symbiodiniaceae sequence datasets were downloaded: *Cladocopium goreaui* (formerly Clade C, type C1) and *Fugacium kawagutii* (formerly Clade F) whole-genome sequences (*79*) from http://symbs.reefgenomics.org/download/; *Breviolum minutum* (formerly Clade B) transcriptome, assembled genome, and genome-predicted transcripts from http://marinegenomics.oist.jp/ (project ID 21) (*80*); and all *Symbiodinium* (tax ID 2949) transcriptomes and EST sequences (*81*–*83*) from GenBank and from http://medinalab.org/zoox/. The Symbiodiniaceae-positive matches, of which *C. goreaui* hits comprised more than 90% of the positive reads, were eliminated and the remaining unmapped reads were trimmed using TrimeGalore software (version 0.4.0) (www.bioinformatics.babraham.ac.uk/projects/trim_galore/) to remove adapters, primers, and low-quality bases. All reads were then merged and submitted to Trinity software (version 2.8.4) (*84*) for transcriptome assembly using the default parameters, yielding 1,361,525 Trinity transcripts (N50 = 1272; median contig length = 373). The “align_and_estimate_abundance.pl” script included with the Trinity software was used to map the reads from each sample back to the assembled transcriptome and calculate sample-specific abundance for each transcript [using the RNA-seq by expectation-maximization (RSEM) method] (*85*). To reduce the number of erroneous isoforms, the highest expressed isoform per gene was retained (N50 = 693; median contig length = 327). In addition, all transcripts with a transcripts per million (TPM) value <9 across any sample were filtered out and excluded from further analysis, yielding 115,303 transcripts. Putative coding regions were extracted from the transcriptome assemblies using the TransDecoder software (www.transdecoder.sourceforge.net), with a minimum length of 100 amino acids, providing all the coding sequence and proteins from the assembly. The *E. paradivisa* library contained 54,053 ORF encoding transcripts.

Annotations for ortholog and paralog genes were assigned in two ways. For orthologs, a reciprocal blast of the newly identified proteome was performed against *Homo sapiens* and UniRef50 database using the Protein Basic Local Alignment Search Tool (BLASTP) [National Center for Biotechnology Information (NCBI)]. For paralogs, annotations were assigned by blasting the remaining unannotated transcripts against *H. sapiens*, UniRef50, UniProtKB/Swiss-Prot, and NR databases using BLASTP (NCBI). Filtering was applied to the BLASTP results to increase the certainty of obtaining true homologs. Filtering parameters were set at an *e* value threshold of 5 × 10^−5^, >30% alignment identity, and >70% query coverage. In total, we were able to assign annotation to 27,080 (50%) transcripts, of which 4457 have orthologous relationships.

### Rhythmicity analysis

Rhythmicity in transcript expression was identified using the RAIN package for R (*24*). The RAIN algorithm was run separately for each *E. paradivisa* morph in each light condition (LDsym, DDsym, LDapo, and DDapo) as one dataset. For each dataset, all replicates (*n* = 3) for each time point were analyzed as regular time series to detect daily and tidally oscillating transcripts. For both the diel and tidal analyses, we only looked for transcripts with a precise 24- or 12-hour period and not a range (e.g., 10 to 14 or 20 to 28 hours). To account for multiple testing, transcripts with a Benjamini-Hochberg–corrected RAIN *P* value < 0.01 were considered confident cyclers (*24*). To perform clustering of the rhythmic transcripts based on GMMs, we used the Mclust function on the R mclust package (V.5.2) (*86*). The analyses were run separately for the 24- and 12-hour oscillating transcripts from each of the 48-hour sampled subgroups (LDsym, DDsym, and LDapo). Heatmaps were generated using the heatmap.2 function on the R package gplots (V.2.17.0). Venn diagrams were generated using the web tool Venn diagram (http://bioinformatics.psb.ugent.be/webtools/Venn/). Expression plots were generated using GraphPad Prism (V.9.1.0). Functional enrichment analyses of annotated rhythmic transcripts were conducted using Metascape (*25*). By default, all input annotations were converted into their human orthologs for the enrichment analysis. A *P* value of 0.01 was used as the threshold for statistical significance.

## Notes

View/request a protocol for this paper from *Bio-protocol*.
