# Supplementary material for: Temporal gene expression patterns in the coral Euphyllia paradivisa reveal the complexity of biological clocks in the cnidarian-algal symbiosis
Source: Sci Adv. 2022 Sep 16;8(37):eabo6467. doi: 10.1126/sciadv.abo6467 (PMC9481131; doi:10.1126/sciadv.abo6467)
Supplement: Supplementary file 1 — Figs. S1 and S2 Table S1 [file sciadv.abo6467_sm.pdf]

Science Advances
AAAS

Supplementary Materials for

Temporal gene expression patterns in the coral *Euphyllia paradivisa* reveal the complexity of biological clocks in the cnidarian-algal symbiosis

Mieka Rinsky *et al.*

Corresponding author: Mieka Rinsky, miekari9@gmail.com; Oren Levy, oren.levy@biu.ac.il

Sci. Adv. **8**, eabo6467 (2022)
DOI: 10.1126/sciadv.abo6467

The PDF file includes:

Figs. S1 and S2
Table S1
Legend for table S2
Legends for data S1 to S3

Other Supplementary Material for this manuscript includes the following:

Table S2
Data S1 to S3

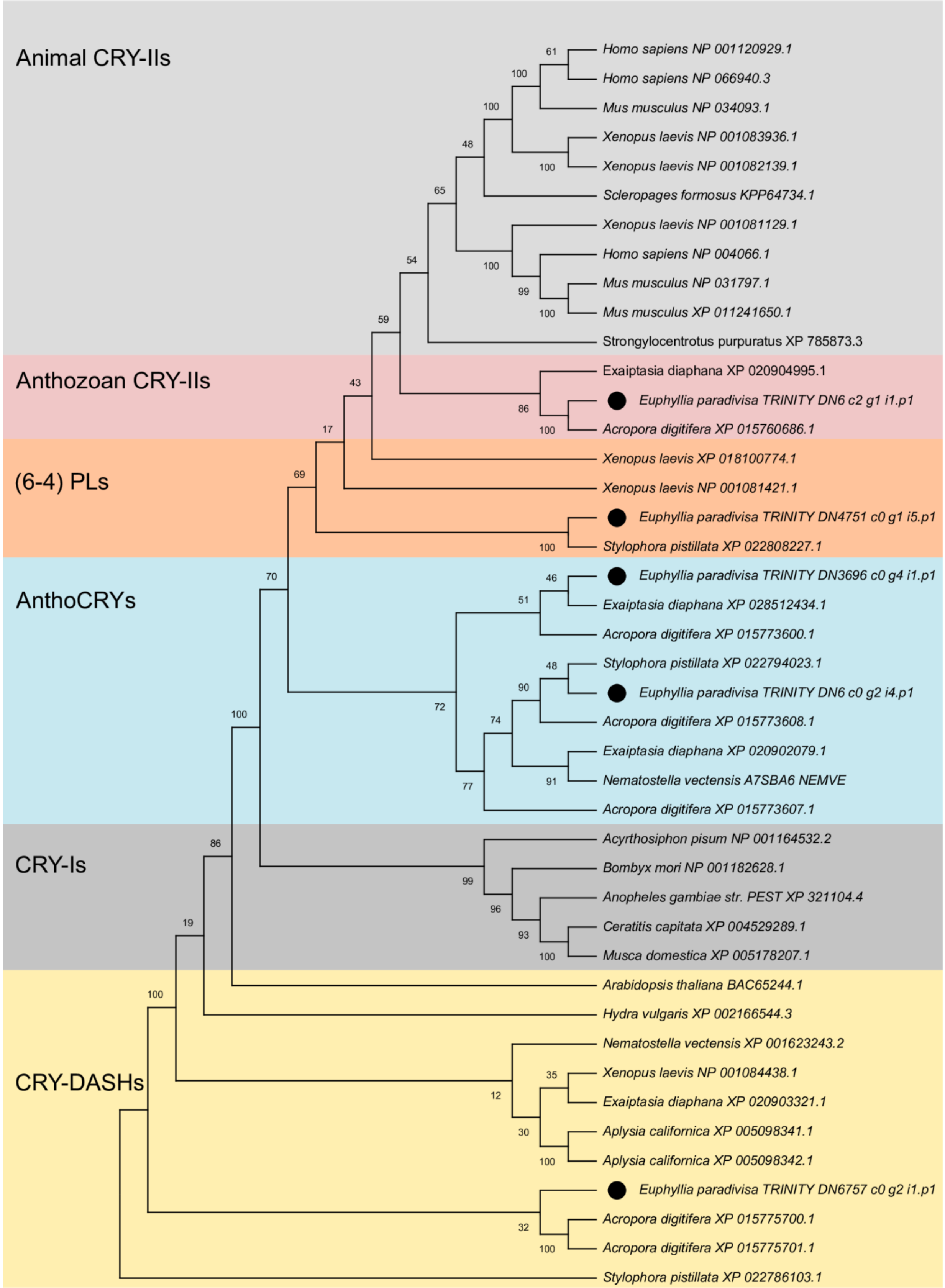
Animal CRY-IIs
Homo sapiens NP 001120929.1
Homo sapiens NP 066940.3
Mus musculus NP 034093.1
Xenopus laevis NP 001083936.1
Xenopus laevis NP 001082139.1
Scleropages formosus KPP64734.1
Xenopus laevis NP 001081129.1
Homo sapiens NP 004066.1
Mus musculus NP 031797.1
Mus musculus XP 011241650.1
Strongylocentrotus purpuratus XP 785873.3
Anthozoan CRY-IIs
Exaiptasia diaphana XP 020904995.1
Euphyllia paradivisa TRINITY DN6 c2 g1 i1.p1
Acropora digitifera XP 015760686.1
(6-4) PLs
Xenopus laevis XP 018100774.1
Xenopus laevis NP 001081421.1
Euphyllia paradivisa TRINITY DN4751 c0 g1 i5.p1
Stylophora pistillata XP 022808227.1
AnthoCRYs
Euphyllia paradivisa TRINITY DN3696 c0 g4 i1.p1
Exaiptasia diaphana XP 028512434.1
Acropora digitifera XP 015773600.1
Stylophora pistillata XP 022794023.1
Euphyllia paradivisa TRINITY DN6 c0 g2 i4.p1
Acropora digitifera XP 015773608.1
Exaiptasia diaphana XP 020902079.1
Nematostella vectensis A7SBA6 NEMVE
Acropora digitifera XP 015773607.1
CRY-Is
Acyrthosiphon pisum NP 001164532.2
Bombyx mori NP 001182628.1
Anopheles gambiae str. PEST XP 321104.4
Ceratitis capitata XP 004529289.1
Musca domestica XP 005178207.1
CRY-DASHs
Arabidopsis thaliana BAC65244.1
Hydra vulgaris XP 002166544.3
Nematostella vectensis XP 001623243.2
Xenopus laevis NP 001084438.1
Exaiptasia diaphana XP 020903321.1
Aplysia californica XP 005098341.1
Aplysia californica XP 005098342.1
Euphyllia paradivisa TRINITY DN6757 c0 g2 i1.p1
Acropora digitifera XP 015775700.1
Acropora digitifera XP 015775701.1
Stylophora pistillata XP 022786103.1

Fig. S1. Phylogenetic analysis of *Cry* genes. Maximum Parsimony tree, bootstrap method with 1,000 iterations, aligned by MUSCLE codons generated using the MEGA X software version 10.0.5. Reference sequences were chosen based on Supplementary Figure 5 in Gornik et al. 2021 (*27*). Euphyllia paradivisa proteins are marked with a solid circle.

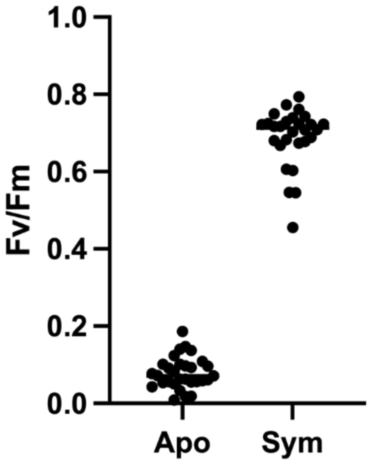

Fig. S2. Photosynthetic quantum yield (*Fv*/*Fm*) of *Euphyllia paradivisa* symbionts. Measurements of representative symbiotic (Sym) and aposymbiotic (Apo) *E. paradivisa* polyps ($n = 80$) were taken prior to the experiment using a diving PAM (pulse amplitude modulator) fluorometer (Walz).

Gene name	Subgroup	Period (hour)	RAIN *P*-value
Cry1	**LDsym**	24^{***}	1.97E-16
	**DDsym**	24^{***}	2.62E-05
	**LDapo**	24^{***}	7.40E-14
	**DDapo**	24^{***}	5.29E-05
Cipc-like	**LDsym**	24^{***}	3.8E-17
	**DDsym**	24^{***}	0.000000711
	**LDapo**	24^{***}	1.88E-13
	**DDapo**	24^{**}	0.001187469
Hlf	**LDsym**	24^{***}	1.05E-22
	**DDsym**	24^{***}	2.99E-06
	**LDapo**	24^{***}	8.10E-21
	**DDapo**	24^{***}	0.000129121
Tef	**LDsym**	24^{***}	1.93E-15
	**DDsym**	24^{**}	0.002730665
	**LDapo**	24^{***}	3.70E-16
	**DDapo**	24^{***}	0.00012925
Hebp2	**LDsym**	24^{***}	3.89E-10
	**DDsym**	24^{***}	9.40E-05
	**LDapo**	24^{***}	1.79E-17
	**DDapo**	24^{***}	0.00084787
Clock	**LDsym**	24^{***}	6.93E-17
	**DDsym**	24^{*}	0.033
	**LDapo**	24^{***}	3.77E-14
	**DDapo**	24^{ns}	0.349
Cry2	**LDsym**	24^{***}	2.52E-08
	**DDsym**	24^{ns}	0.056
	**LDapo**	24^{***}	6.85E-11
	**DDapo**	24^{ns}	0.602
Casein kinase 1	**LDsym**	24^{***}	4.05E-08
	**DDsym**	24^{ns}	0.085
	**LDapo**	24^{***}	6.47E-07
	**DDapo**	24^{ns}	0.186
Cry-DASH	**LDsym**	24^{***}	4.98E-09
	**DDsym**	24^{ns}	0.339
	**LDapo**	24^{***}	4.98E-09
	**DDapo**	24^{*}	0.011

Table S1. RAIN results for candidate circadian clock genes of E. paradivisa subgroups.
*Asterisks indicate the power of significance: $*P < 0.05$; $**P < 0.01$; $***P < 0.001$; ^{ns}not significant.
†Exact Benjamini-Hochberg corrected RAIN *P*-values.

Table S2. 24-h to 12-h period alternations.

List of 59 E. paradivisa annotated transcripts showing 24-h to 12-h period alternations between symbiotic and aposymbiotic morphs.

Data S1. Annotation and expression patterns of rhythmic transcripts in each subgroup.

Each subgroup: **A**) LDsym24, **B**) DDsym24, **C**) LDapo24, **D**) DDapo24, **E)** LDsym12, **F**) Dsym12, **G**) LDapo12, **H**) DDapo12.
Data S1I. Eight-way Venn diagram analysis.

Data S2. Gaussian mixture models (GMM) clustering results.

Data S2A. Results for rhythmic transcripts of the LDapo24, LDsym24, and DDsym24 subgroups.
Data S2B. Results for rhythmic transcripts of the LDapo12, LDsym12, and DDsym12 subgroups.

Data S3. Functional enrichment analysis.

Data S3A. Analysis of the rhythmic genes with a precise 24-h period of each E. paradivisa subgroup found in Cluster 1.
Data S3B. Analysis of the rhythmic genes with a precise 24-h period of each E. paradivisa subgroup found in Cluster 2.
Data S3C. Analysis of the rhythmic genes with a precise 24-h period of each E. paradivisa subgroup found in Cluster 3.
Data S3D. Analysis of the rhythmic genes with a precise 24-h period of each E. paradivisa subgroup found in Cluster 4.
Data S3E. Analysis of the rhythmic genes with a precise 12-h period of each E. paradivisa subgroup found in Cluster 1.
Data S3F. Analysis of the rhythmic genes with a precise 12-h period of each E. paradivisa subgroup found in Cluster 2.
Data S3G. GO analysis of rhythmic genes showing 12-h to 24-h period alternations between sym to apo E. paradivisa
Data S3H. GO analysis of rhythmic genes showing 24-h to12-h period alternations between sym to apo E. paradivisa
